# Predictive ability of C-reactive protein for stroke

**Published:** 2016

**Authors:** Alijan Ahmadi-Ahangar

**Affiliations:** 1 Mobility Impairment Research Center, Babol University of Medical Sciences, Babol, Iran.

Stroke is an important cause of morbidity and mortality as well as functional impairment particularly in the elderly people (1). Prediction of stroke outcome in high risk patients by appropriate marker and application of preventive measures may delay or prevent development of irreversible brain damage and subsequent functional impairment (2). Development of stroke is the result of longstanding vascular inflammation, plaque rupture, thrombosis and subsequent brain ischemia or infarction (3). Among the several markers of inflammation, serum C-reactive protein (CRP) is of particular importance. This marker can be used not only for the detection of inflammatory state and evaluation of treatment (4, 5) but also for the prediction of future development of atherosclerotic diseases including stroke and cardiovascular disease (6). Bakhsayesh et al. in one issue of Caspian Journal of Internal Medicine have investigated the ability of serum CRP and white blood cell (WBC) as markers of inflammation in predicting the outcome of acute ischemic stroke. The results indicated that serum CRP levels >10.5 m/l predicted mortality of stroke over three months of follow-up duration at sensitivity and specificity of 75% and 63.8%, respectively. In addition, serum CRP > 8.5mg/l differentiated stroke patients with and without poor prognosis at sensitivity of 73.1%, and specificity of 69.4% whereas WBC showed predictive ability for stroke (7).

Several previously published studies found an association between high serum CRP level and development of stroke (6, 8-10). Rooco et al. found an independent association between high serum CRP level with mortality and intercerebral hemorrhage after thrombolysed stroke (2). 

Similarly, Ridker et al., found a positive correlation between serum hsCRP and severity of stroke (10). In a prospective longitudinal study of 10456 healthy men by Jimenez et al, baseline serum CRP > 3 mg/ml was associated with increased risk of incident stroke by 40% as compared with CRP<1 mg/l over a 15-year follow-up period The risk was greater in hypertensive rather than normotensive men (8). In another study by Moon et al., serum hsCRP> 0.31 mg/dl predicted development of cerebrovascular and cardiac events after percutaneous coronary intervention over a mean follow-up period of 28.5 months (6). The results of a systematic review of 12 prospective observational studies revealed an independent association of baseline CRP with excessive risk of ischemic stroke but not hemorrhagic stroke (9). Furthermore, carotid atherosclerosis has been shown to be associated with serum CRP (11) and increasing serum CRP in patients with internal carotid stenosis may indicate ischemic events (12). These findings indicate an association between serum CRP and a stroke suggesting a link between inflammatory process and outcome in patients who experienced a stroke. Nonetheless, the results of studies which assessed the relationship between serum CRP and stroke should be considered with limitations. Several common clinical conditions such as hypertension, diabetes, hyperlipidemia, obesity, metabolic syndrome, smoking, vitamin D deficiency, high parity and low bone mineral density are linked to stroke, or inflammation and these factors are also prevalent in the general populations (1, 5, 12-23). These factors may increase the risk of stroke and affect the outcome regardless of inflammatory state. Many chronic clinical diseases that are also associated with low grade systemic inflammation (15, 16, 22) usually coexisted in elderly subjects who are at greater risk of stroke and thus provide further predisposition for stroke. In addition, asymptomatic latent and undiagnosed local or systemic infection may be a cause of increased serum CRP and confound the results. These observations suggest appropriate application of statistical analysis test to determine the extent of contribution of CRP in the development of stroke.

## Citation:

Ahmadi-Ahangar A. Predictive ability of C-reactive protein for stroke. Caspian J Intern Med 2016; 7(3): 151-152.
